# Ixodid ticks and zoonotic tick-borne pathogens of the Western Balkans

**DOI:** 10.1186/s13071-023-06116-1

**Published:** 2024-01-31

**Authors:** Naida Kapo, Ivana Zuber Bogdanović, Ema Gagović, Marina Žekić, Gorana Veinović, Ratko Sukara, Darko Mihaljica, Bojan Adžić, Përparim Kadriaj, Aleksandar Cvetkovikj, Igor Djadjovski, Aleksandar Potkonjak, Enkelejda Velo, Sara Savić, Snežana Tomanović, Jasmin Omeragić, Relja Beck, Adnan Hodžić

**Affiliations:** 1 Department of Veterinary Clinical Sciences, Faculty of Veterinary Medicine, University of Sarajevo, Sarajevo, Bosnia and Herzegovina; 2 Diagnostic Veterinary Laboratory, Podgorica, Montenegro; 3 Department for Bacteriology and Parasitology, Laboratory for Parasitology, Croatian Veterinary Institute, Zagreb, Croatia; 4 Scientific Veterinary Institute “Novi Sad”, Novi Sad, Serbia; 5 Institute for Medical Research, National Institute of Republic of Serbia, University of Belgrade, Belgrade, Serbia; 6 Vector Control Unit, Department of Epidemiology and Control of Infectious Diseases, Institute of Public Health, Tirana, Albania; 7 Veterinary Institute, Faculty of Veterinary Medicine, Ss. Cyril and Methodius University in Skopje, Skopje, North Macedonia; 8 Department of Veterinary Medicine, Faculty of Agriculture, University of Novi Sad, Novi Sad, Serbia; 9 Department of Microbiology and Ecosystem Science, Centre for Microbiology and Environmental Systems Science (CMESS), University of Vienna, Vienna, Austria

**Keywords:** Hard ticks, Tick-borne diseases, Western Balkans, Zoonoses

## Abstract

Ixodid ticks are distributed across all countries of the Western Balkans, with a high diversity of species. Many of these species serve as vectors of pathogens of veterinary and medical importance. Given the scattered data from Western Balkan countries, we have conducted a comprehensive review of available literature, including some historical data, with the aim to compile information about all recorded tick species and associated zoonotic pathogens in this region. Based on the collected data, the tick fauna of the Western Balkans encompasses 32 tick species belonging to five genera: *Ixodes*, *Haemaphysalis*, *Dermacentor*, *Rhipicephalus* and *Hyalomma*. A range of pathogens responsible for human diseases has also been documented, including viruses, bacteria and parasites. In this review, we emphasize the necessity for integrated surveillance and reporting, urging authorities to foster research by providing financial support. Additionally, international and interdisciplinary collaborations should be encouraged that include the exchange of expertise, experiences and resources. The present collaborative effort can effectively address gaps in our knowledge of ticks and tick-borne diseases.

**Graphical Abstract:**

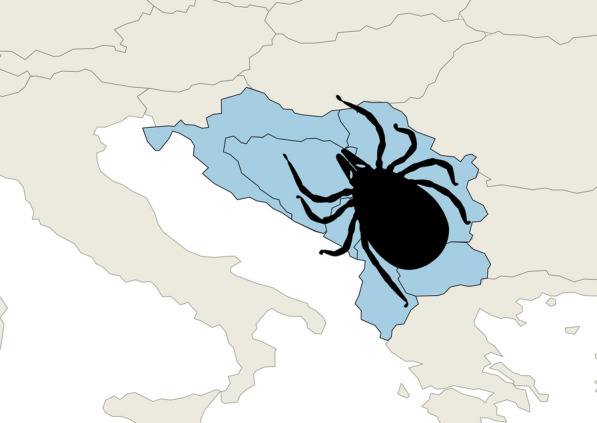

## Background

Ixodid ticks are obligate hematophagous ectoparasites that feed on the blood of virtually all terrestrial vertebrates, including humans. However, their importance resides in their capacity to transmit a multitude of microbial (viruses, bacteria) and parasitic (protozoa, helminths) disease-causing agents [1, 2]. The blood-feeding habit and exploitation of different hosts in each active developmental stage enhance the tick’s potential to maintain and transmit pathogens, which makes them some of the most efficient vectors of diseases affecting both human and animal health [2]. In temperate climate regions, ticks are responsible for more human and animal infections than any other blood-feeding arthropod, transmitting pathogens such as spotted fever group (SFG) rickettsiae and piroplasms, *Borrelia burgdorferi* sensu lato (*B burgdorferi* s.l.), tick-borne encephalitis virus (TBEV) and Crimean-Congo hemorrhagic fever virus (CCHFV) [3]. In recent decades, there has been a notable increase in the occurrence, variety, and geographical distribution of tick-borne diseases (TBDs). This trend is closely aligned with the heightened scientific awareness and the impact of human activities on both living organisms and abiotic components of natural ecosystems [4–6]. Global warming, socio-demographic factors, deforestation, and reduction of wildlife habitats inducing migration of wildlife populations are believed to be the main underlying factors facilitating the geographic expansion of ticks and tick-borne pathogens (TBPs) [7, 8].

The region of Western Balkans covers an area of about 208,000 km^2^ and includes Albania, Bosnia and Herzegovina, Croatia, Montenegro, North Macedonia (N. Macedonia), and Serbia. This area has a mix of different climates ranging from Mediterranean climate to moderately continental, and it is characterized by highly variable weather conditions, including humid, cold winters and dry, hot summers [9]. The spatio-temporal distribution of ixodid ticks is mainly determined by climatic conditions, due to the majority of their life cycle occurring in the environment. Five ixodid tick genera, namely *Ixodes* (*I.*), *Haemaphysalis* (*Hae.*), *Dermacentor* (*D.*), *Rhipicephalus* (*Rh.*), and *Hyalomma* (*Hy.*), have been found in the Western Balkans. While, among 37 ixodid tick species described so far in the Mediterranean part of Europe, 32 have been documented in the Balkans alone [3]. Moreover, Western Balkan is also considered a hotspot for neglected infections of poverty [10] due to the new pathogen species, strains, or genetic variants that are constantly being recognized in the ticks and their vertebrate hosts (e.g. [11, 12]).

This review aims to provide exhaustive information, including some historical records, on the occurrence and distribution of ixodid ticks and zoonotic TBPs recorded across each Western Balkan country (Tables 1, 2, and 3). Some tick species were originally reported under different names (synonyms or misidentification), therefore current names according to the most recent nomenclature are given [3]. We also systematically revise and analyze the past and the current situation in the region and highlight future research priorities to encourage studies on this important topic and raise awareness among parasitologists, veterinarians, and physicians.

**Table 1:** A list of ixodid tick species reported in the Western Balkan countries

Country	Ixodid tick species^a^	References
Albania	*I. ricinus*, *Hae. inermis, Hae. punctata*, *Hae. sulcata*, *D. marginatus*, *Rh. bursa*, *Rh. sanguineus*, *Rh. turanicus*, *Rh. annulatus*, *Hy. marginatum*, *Hy. scupense*, *Hy. excavatum*	[3, 17, 32, 190–192]
Bosnia and Herzegovina	*I. ricinus*, *I. hexagonus*, *I. canisuga*, *I. vespertilionis*, *Hae. inermis, Hae. punctata*, *Hae. sulcata*, *Hae. concinna*, *D. marginatus*, *D. reticulatus*, D. silvarum, *Rh. bursa*, *Rh. sanguineus*, *Rh. annulatus*, *Hy. marginatum*, Hy. rufipes, *Hy. aegyptium*, *Hy. scupense*, *Hy. excavatum*	[3, 13, 14, 16, 34–36, 144, 193–206]
Croatia	*I. ricinus*, *I. hexagonus*, *I. canisuga*, *I. vespertilionis*, *I. kaiseri*, *I. frontalis*, *I. arboricola*, *I. gibbosus*, *I. trianguliceps*, *Hae. inermis, Hae. punctata*, *Hae. sulcata*, *Hae. concinna*, *Hae. parva*, *Hae. erinacei*, *D. marginatus*, *D. reticulatus*, *D. silvarum*, *Rh. bursa*, *Rh. sanguineus*, *Rh. turanicus*, Rh. annulatus, *Hy. marginatum*, *Hy. scupense*, Hy. dromedarii	[3, 14, 146, 207–215]
Montenegro	*I. ricinus*, *I. simplex*, *I. vespertilionis*, *Hae. inermis, Hae. punctata*, *Hae. sulcata*, *D. marginatus*, *D. reticulatus*, *D. silvarum*, *Rh. bursa*, *Rh. sanguineus*, *Rh. annulatus*, *Hy. marginatum*, *Hy. rufipes*, *Hy. scupense*, *Hy. dromedarii*	[3, 16, 34, 116]
North Macedonia	*I. ricinus*, *I. simplex, Hae. punctata*, *Hae. sulcata*, *Hae. inermis*, *D. marginatus*, *D. reticulatus*, *D. silvarum*, *Rh. bursa*, *Rh. sanguineus*, *Rh. annulatus*, *Hy. marginatum*, *Hy. rufipes*, *Hy. Scupense, Hy. excavatum*	[3, 123–127, 130, 145]
Serbia	*I. ricinus*, *I. hexagonus*, *I. canisuga*, *I. vespertilionis*, *I. kaiseri*, *I. simplex*, *I. laguri*, *I persulcatus*, *Hae. inermis, Hae. punctata*, *Hae. sulcata*, *Hae. concinna*, *Hae. leporispalustris*, *D. marginatus*, *D. reticulatus*, *D. silvarum*, *Rh. bursa*, *Rh. sanguineus*, *Rh. annulatus*, *Hy. marginatum*, Hy. rufipes, *Hy. scupense*, *Hy. excavatum*, *Hy. dromedarii*	[3, 137–145, 216]

Underlined tick species are not native to the Western Balkan countries and are most likely incorrectly described in the original papers

^a^Ixodid tick genera and their abbreviations: *Ixodes* (*I.*), *Haemaphysalis* (*Hae.*), *Dermacentor* (*D.*), *Rhipicephalus* (*Rh.*), and *Hyalomma* (*Hy.*), have been found in the Western Balkans

**Table 2:** Zoonotic tick-borne pathogens affecting animals in the Western Balkans

Country	Pathogen	Host^a^	Prevalence (%)	Method	References
Albania	*Anaplasma* spp./*Ehrlichia* spp.	*Rh. bursa Rh. sanguineus*,	12.5–14.3	PCR, RLBH	[17]
	*E. canis*	*Rh. sanguineus*	3.6	PCR	[17]
	*Rickettsia* IRS3	*Rh. bursa*, *Hy. marginatum*	6.2–43.3	PCR	[17]
	*R. helvetica*	*Rh. sanguineus*, *Rh. bursa*	3.6–6.2	PCR	[17]
	*R. conorii*	*Rh. sanguineus*, *Rh. bursa*, *Hy. marginatum*	3.1–26.6	PCR	[17]
	*C. burnetii*	Cattle, sheep, goat	9.1	ELISA	[22]
	*B. garinii*	Dog	49.4	ELISA	[21]
	CCHFV	*Rh. bursa*, *Hy. marginatum*, cattle, sheep, goat	0.03–88.3	IFAT, ELISA, PCR	[27–32]
Bosnia and Herzegovina	*B. divergens*	Cattle, *I. ricinus*	n/a	Blood smear, PCR-Seq	[36, 37]
	*A. phagocytophilum*	*D. reticulatus*, dog	1.1–4.9	PCR-Seq, mf qPCR	[40, 44]
	*A. phagocytophilum*	Dog	24	qPCR	[41]
	*A. phagocytophilum/A. platys*	Dog	0.5	qPCR	[41]
	*A. phagocytophilum/A. platys*	Dog	20.4	SNAP 4Dx	[41]
	*E. canis/E. ewingii*	Dog	0.2	SNAP 4Dx	[41]
	*R. monacensis*	*I. ricinus*	1.1	PCR-Seq	[44]
	*R. helvetica*	*I. ricinus*	5.7	PCR-Seq	[44]
	*R. raoultii*	*D. reticulatus*	5.7	PCR-Seq	[44]
	*R. slovaca*	*D. reticulatus*, *D. marginatus*	8.0	PCR-Seq	[44]
	*F. tularensis* subsp. *holarctica*	*D. marginatus*	1.1	PCR-Seq	[44]
	TBEV (Bosnia lineage)	*I. ricinus*	n/a	Isolation, PCR, genome seq	[45, 227]
	CCHFV	*Hy. marginatum*^b^, cattle, sheep	0.4^a^–15.0	IFAT, ELISA, RT-PCR	[46, 47]
Croatia	*B. microti*	Rodent	3.3–14.0	RT-PCR, PCR-Seq	[79, 221]
	*Anaplasma* spp.	Dog, rodent	4.5–6.2 (dogs), 1.65 (rodents)	SNAP 4Dx, PCR	[49, 70, 71]
	*A. phagocytophilum*	*I. ricinus*, various animals	< 38.0	IFAT, PCR-Seq	[50, 75, 77]
	*A. phagcytophilum*	Horse	Case report	PCR-Seq	[75, 220]
	*A. phagocytophilum*	Dog	0.3–10.5%	PCR-Seq	[50, 72]
	*Ehrlichia* spp.	Rodent	0.8	PCR-Seq	[49]
	*E. canis*	Dog	0.46	SNAP 4Dx	[70, 71]
	*N. mikurensis*	Dog	4.6	PCR-Seq	[50]
	*Rickettsia* spp.	Rodent	1.8–4.3	RT- PCR	[63]
	SFG Rickettsiae	Ticks from sheep, goat, cattle	1.6	PCR-Seq	[65]
	*R. helvetica*	*I. ricinus, D. reticulatus*	6.2–10.0	PCR-Seq	[48, 68]
	*R. raoultii*	*I. ricinus*	0.08	PCR	[48]
	*R. slovaca*	*D. reticulatus, D. marginatus*	2.0–60.0	PCR-Seq	[48, 65, 66, 68]
	*R. conorii*	Dog, cattle, goat, sheep, ticks	23–51.0	IFAT, PCR-Seq	[61, 66, 68, 145]
	*R. aeshlimanii*	*Hy. marginatum*	26.1–64.7	PCR-Seq	[65, 66]
	*R. rhipicephali*	*Rh. turanicus*	9.1	PCR-Seq	[66]
	*R. felis*-like	*Hae. sulcate* from sheep, goats	19.6–26.0	PCR-Seq	[67]
	*F. tularensis*	Rodent	0.8	PCR-Seq	[49]
	*C. burnetii*	Dog, cattle, sheep, goat, brown bear, rodents	< 16.4	CFT, IFAT, PCR-Seq	[49, 61, 222]
	*B. burgdorferi* s.l	*I. ricinus*, Dog	0.4–0.7 (dogs)	SNAP 4Dx, IFAT	[70, 71]
	*B. afzelii*	*I. ricinus*, rodent	2.0–21.5	PCR, PCR–RFLP, PCR-Seq	[49, 83, 91]
	*B. garinii*	*I. ricinus*	4.0	PCR-Seq	[223]
	*B. burgdorferi* s.s	*I. ricinus*	0.3–0.8	PCR-Seq, RLBH	[69, 223]
	*B. valaisiana*	*I. ricinus*	0.5–12.0	PCR-Seq, RLBH	[69, 223]
	*B. lustaniae*	*I. ricinus*	0.6	PCR-Seq	[69]
	*B. spielmanii*	*I. ricinus*	0.06	PCR-Seq	[69]
	*B. miyamotoi*	Rodent	3.7	PCR-Seq	[48, 49]
	TBEV	Ticks, animals	< 39.0	PCR, PCR-Seq, RT-PCR	[63, 115]
	BHAV	Bears, sheep	68.0 (bears), 100 (sheep)	HI, ELISA	[94, 222]
Montenegro	*E. canis*	Dog	19.3	IFAT, ELISA	[119]
	*R. conorii*	Dog	73.4	IFAT, ELISA	[119]
	*C. burnetii*	Sheep, dog	1.2–5.0	IFAT	[119, 120]
North Macedonia	*E. canis*	Dog	18.7–45.0	ELISA, PCR	[132, 133]
Serbia	CCHFV	Tick, cattle, sheep, goat	14.6–80.0	ELISA	[130, 131]
	*B. microti*	*I. ricinus*, dog	1.4–1.9	PCR-Seq	[161, 165–167]
	*B. venatorum* *Anaplsma spp.*	*I. ricinus* Ticks	2.8 19.35	PCR-Seq PCR	[165] [166]
	*A. phagocytophilum*	*I. ricinus*, *D. reticulatus*, *Hae. concinna*, dog, golden jackal	0.9–15.5	IFAT, PCR-Seq, mf qPCR	[158, 161, 163, 165, 166, 174, 176, 178, 179]
	*N. mikurensis*	*I. ricinus*	4.2	PCR-Seq	[12, 165, 166]
	*E. canis*	Dog	Case report, 16%	IFAT, PCR-Seq	[152, 180]
	*R. monacensis*	*I. ricinus*	14.8–22.5	PCR-Seq, mf qPCR	[165, 166, 172–175]
	*R. helvetica*	*I. ricinus*	7.4–54.0	PCR-Seq, mf qPCR	[172–175]
	*R. raoultii*	*D. reticulatus*	5.6	PCR-Seq, mf qPCR	[165, 172]
	*R. conorii*	*Rh. sanguineus*, Dog	44.8	IFAT, PCR-Seq	[176, 177]
	*R. massiliae*	*Rh. sanguineus*	0.8	PCR-Seq	[165]
	*F. tularensis*	*I. ricinus*	3.8	PCR/RFLP	[159]
	*C. burnetii*	*I. ricinus*, *D. reticulatus*, *Hae. concinna*, *Rh. Sanguineus*	3.8–62.9	PCR-Seq	[174, 183]
	*B. burgdorferi* s.l	*I. ricinus*, dog, rodent	8.1–54.2	DF, IFAT, ELISA, WB, PCR	[146, 147, 150–152, 154–156, 174, 176]
	*B. afzelii*	*I. ricinus*	7.0–75.0	Isolation, PCR-Seq, PCR/RFLP, RT-PCR	[151, 152, 158–160, 178]
	*B. garinii*	*I. ricinus*, *D. reticulatus*, red fox	0.8–4.9	Isolation, PCR-Seq, PCR/RPLF	[12, 152, 158–162, 178]
	*B. burgdorferi* s.s	*I. ricinus*, red fox	0.8–22.2	PCR-Seq, PCR/RFLP	[12, 152, 159, 178]
	*B. valaisiana*	*I. ricinus*	1.4–3.8	Isolation, PCR-Seq, PCR/RFLP, mf qPCR	[158, 160–163, 178]
	*B. lusitaniae*	*I. ricinus*, red fox	1.6–11.3	Isolation, PCR-Seq, PCR/RFLP, mf qPCR	[12, 151, 158, 160–163, 178]
	*B. bavariensis*	*I. ricinus*	2 isolates	Isolation, PCR-Seq	[162]
	*B. miyamotoi*	*I. ricinus*	1.6–6.0	PCR-Seq, mf qPCR	[160, 163]
	TBEV (EU subtype)	Horse, cattle, roe deer, wild boar, dog	2.5–17.5	Isolation, ELISA, PCR-Seq	[184, 185]

*CFT* Complement fixation test, *DF* dark field microscopy, *ELISA* enzyme-linked immunosorbent assay, *HI* hemagglutination inhibition assay, *IFAT* indirect immunofluorescence test, *mf qPCR* microfluidic real-time PCR, *PCR-RFLP* restriction fragment length polymorphism-PCR, *PCR-Seq* PCR followed by DNA sequencing, *qPCR* real-time PCR, *RLBH* reverse line blot hybridization, *RT-PCR* reverse transcription PCR,* s.l.* sensu lato,* s.s.* sensu stricto, *WB* western blot

^a^*D. Dermacentor*, *Hae. Haemaphysalis*, *Hy. Hyalomma*, *I Ixodes*, *Rh. Rhipicephalus*

^b^Tick pool

**Table 3:** Zoonotic tick-borne pathogens affecting humans in the Western Balkans

Country	Pathogen	Host	Prevalence (%)	Method	References
Albania	*Rickettsia* spp.	Human	2.9	IFAT, PCR	[26]
	*B. burgdorferi* s.l	Human	81.8	ELISA	[20, 21]
	CCHFV	Human	38.2	IFAT, ELISA, PCR	[26]
Bosnia and Herzegovina	*R. conorii*	Human	1.6–1.7	CFT, IFAT	[42]
	*C. burnetii*	Human	19.0–22.4	CFT, IFAT	[42]
	*B. burgdorferi* s.l	*Ioxides ricinus,* human	66.7 (ticks), > 50 cases (humans)	IFAT, ELISA, WB, PCR-Seq	[43, 216–218]
Croatia	*Babesia divergens*	Human	Case report	Biopsy, staining	[78]
	*B. microti*	Human	0.98	IFAT, PCR-Seq	[48, 77]
	*E. chaffeensis*	Human	4.9	IFAT	[77]
	*A. phagocytophilum*	Human	< 38.0	IFAT, PCR	[73, 219]
	*Rickettsia* spp.	Tick, human	7.9–68.1	IFAT, PCR	[48, 60, 77]
	*R. monacensis*	*Ioxides ricinus*, human	21.0 (ticks), 1.5 (human, skin biopsies)	PCR	[48]
	*R. conorii*	Human	4.2/5.0	IFAT, ELISA, PCR	[53, 55, 58, 60, 136]
	*C. burnetii*	Human	170 cases	CFT, IFAT	[58]
	*B. burgdorferi* s.l	*Ixodes ricinus*, human	17.7 (ticks), 9.98–70	ELISA, PCR	[48, 86]
	*B. afzelii*	*Ixodes ricinus*, human	2.0–46.4 (ticks), 80 reports (humans)	PCR, PCR–RFLP, PCR-Seq, RLBH	[48, 83, 223]
	*B. garinii*	*Ixodes ricinus*, human	2.0–4.0; 2.3 (humans)	PCR-Seq, RLBH	[69]
	*B. bavrensis*	Human	2.3	PCR-Seq	[69]
	TBEV	Tick, human	100 cases	CFT, IFAT, ELISA	[114, 224–226]
	BHAV	Human	0–31.5	ELISA, RT-qPCR	[94–99]
Montenegro	*Babesia* spp.	Human	12 cases	Biopsy, blood smear, PCR	[117, 121]
	*E. canis*	Human	64 cases	IFAT, ELISA, WB	[118]
	*R. conorii*	Human	297 cases	IFAT, ELISA, WB	[118]
	*C. burnetii*	Human	158 cases	IFAT	[118]
	*B. burgdorferi* s.l	Human	> 10 cases	IFAT, ELISA, WB, PCR	[117, 121]
North Macedonia	*R. sibirica mongolitimonae*	Human	Case report	IFAT	[136]
	*B. burgdorferi* s.l	Human	2.2–18.8	IFAT, ELISA	[134, 135]
	TBEV	Human	2.2	VNT	[135]
	CCHFV	Human	10 cases	Isolation, IFA, ELISA	[98, 129, 130, 188]
Serbia	*E. chaffeensis*	Human	Two cases	IFAT	[181, 182]
	*Rickettsia* spp.	Human	Two cases	Mf qPCR	[157, 166, 172, 175]
	*R. helvetica*	*Ixodes ricinus*, human	7.4–54.0, two cases (humans)	PCR-Seq, mf qPCR	[163, 166, 172–174]
	*R. slovaca*	*Haemaphysalis* sp. from human	One case	mf qPCR	[171, 172]
	*R. conorii*	Human	1.9–25.0	IFAT	[170]
	*R. aeshlimanii*	*Ixodes ricinus* from humans	3.0	mf qPCR	[163]
	*R. acari*	Human	4.0–10.4	IFAT	[170]
	*R. felis*	*Ixodes ricinus* from humans	4.3	mf qPCR, RT-PCR	[163, 166]
	*Borrelia* spp.	Ticks from humans	19.35	RT-PCR	[166]
	*B. burgdorferi* s.l	Human	8.1–54.2	IFAT, ELISA, WB	[148, 149, 157]
	*B. afzelii*	Human	One case	RT-PCR, mf qPCR	[163]
	TBEV (EU subtype)	*Ixodes ricinus*, human	2.0–6.6 (ticks), 0.3–13.2 (humans)	IFAT, ELISA	[185–187]
	CCHFV	*Ixodes ricinus*, *Hyalomma marginatum*, human	Isolates, case reports (humans)	Isolation, IFAT, PCR	[188, 189]

*CFT* Complement fixation test, *DF* dark field microscopy, *ELISA* enzyme-linked immunosorbent assay, *HI* hemagglutination inhibition assay, *IFAT* indirect immunofluorescence test, *mf qPCR* microfluidic real-time PCR, *PCR-RFLP* restriction fragment length polymorphism PCR, *PCR-Seq* PCR followed by DNA sequencing, *qPCR* real-time PCR, *RLBH* reverse line blot hybridization, *RT-PCR* reverse transcription PCR,* s.l.* sensu lato, *VNT* virus neutralization test, *WB* western blot

## Brief history of ticks and tick-borne pathogens of public health concern in the Western Balkans

The first scientific records of tick occurrence in the Western Balkans date back to the early twentieth century. These early studies on ticks primarily consisted of rare and limited findings of tick species, primarily located on domestic animals, accompanied to some degree by descriptions of their physical characteristics and seasonal patterns. In 1910, Neumann provided the first written record of ixodid tick species in the Western Balkans, reporting the occurrence of *Hy. aegyptium* from cattle in Bosnia and Herzegovina and *Hae. punctata* and *Rh. bursa* in Croatia [13]. According to Muftić [14], tick research in the Republic of N. Macedonia was first conducted by Knuth et al. [15] in 1917. These researchers recorded the presence of *D. reticulatus*, *Rh. bursa*, *Rh. sanguineus* and *Hy. aegyptium* on equines. In the other Balkan countries, investigations commenced later, as documented by Oswald [16]. However, most of these records are written in Balkan-Slavic languages, and as such, they are not available in present-day online repositories nor understandable to non-native researchers, which had led to certain discrepancies in the literature over the years. More intensive systemic research in the Western Balkans was carried out in the 1990s and 2000s. In the period before the 1990s, comprehensive knowledge about the distribution of tick species at the national level was primarily obtained through doctoral or project-based research. However, this approach resulted in fragmented information due to the limited scope of these studies, which often focused on specific tick species, hosts, time frames and/or geographical areas. However, it should be noted that despite these studies covering a relatively small geographical area, the results were highly diverse across countries, likely due to the different climate and topography conditions, as well as the different keys used for the morphological tick determination. These factors often led to misidentification and/or incorrect description of the collected specimens.

In contrast to the research on ticks, investigations on TBPs did not receive the same amount of attention in the past, mostly due to diagnostic challenges and lack of interest within the context of public health. However, the number of publications dealing with TBDs from the Western Balkans has significantly increased in the last 10–15 years after the introduction of molecular diagnostic tools. The consequent changes in tick distribution and the increasing economic, social and public health impact of TBDs are also the reasons for increasing research interest in the region.

Passive surveillance programs are currently being implemented across multiple Western Balkan countries, representing a valuable method for national-level data collection. This approach entails ongoing and systematic monitoring conducted over an extended time, facilitating the comprehensive accumulation of data. However, tick and disease monitoring programs are still inferior to those related to mosquito-borne diseases.

## Albania

### Ticks

The earliest record of tick species in Albania came from Christova et al. [17] in 2003, and since then, 12 species have been documented, all belonging to the category of hard ticks: *I. ricinus*, *Hae. inermis*, *Hae. punctata*, *Hae. sulcata*, *D. marginatus*, *Rh. bursa*, *Rh. sanguineus* sensu lato (*Rh. sanguineus* s.l.), *Rh. turanicus*, *Rh. annulatus* (syn. *Boophilus annulatus*), *Hy. marginatum* (syn. *Hy. plumbeum*), *Hy. scupense* (syn. *Hy. detritum*) and *Hy. excavatum* (Table 1).

### Tick-borne pathogens

The first epidemiological survey on tick-borne bacteria in Albania was conducted by Christova et al. [17]. Overall, in this survey, 90 *Rh. bursa*, *Rh sanguineus* sensu lato and *Hy. marginatum* ticks were collected from cattle in five localities of northern and middle Albania and tested for the presence of *B. burgdorferi* s.l., *Anaplasma/Ehrlichia* and SFG rickettsiae by PCR and reverse line blot hybridization (RLBH) [17]. Four (12.5%) *R. bursa* and 14.3% (4/28) *R. sanguineus* ticks were positive for *Anaplasma*/*Ehrlichia* by RLBH and 44.4% (40/90) of the ticks tested positive for rickettsiae. *Rickettsia helvetica* was identified in 6.2% (2/32) of *Rh. bursa* and in 3.6% (½8) of *Rh. sanguineus* s.l. ticks, while *R. monacensis* (originally described as *Rickettsia* sp. IRS3) was molecularly confirmed in 6.2% (2/32) of *Rh. bursa* and 43.3% (12/30) of *Hy. marginatum*. *Rickettsia conorii*, a causative agent of the Mediterranean spotted fever (MSF), was detected in 26% (7/30) of *Hy. marginatum*, in 10.7% (3/28) of *Rh. sanguineus* s.l. and in 3.1% (1/32) of *Rh. bursa*. Only one tick of *Hy. marginatum* was double infected with *R. conorii* and *R. monacensis*. *Ehrlichia canis* was only detected in 3.6% (1/28) of *Rh. sanguineus* s.l. by PCR [17]. This bacterium primarily infects canids, but it has also been associated with human infection (human monocytic ehrlichiosis) [18].

Three different genotypes of *Anaplasma* species (*A. marginale*, *A. centrale* and *A. ovis*) were detected in 203 samples of spleen obtained from goats, sheep and calves in May–July 2000 in the northern part of Albania, Shkodër district [19]. DNA of *Anaplasma* sp. was detected in 48% (35/73) of the sheep tissue samples, 44% (30/68) of the goat tissue samples and 22.6% (14/62) of the calf tissue samples. *Anaplasma* sp. identified in a single goat sample was 97% identical to the sequences of *A. phagocytophila* [19]*.*

Lyme borreliosis was reported in Albania for the first time in 1982, with 15 out of 50 clinically suspected human patients found to be seropositive [20]. In a later study, conducted between 2011 and 2015, Myrseli et al. [21] reported 11 new cases, with an average of 2.5 cases per year. In the same study, the authors analyzed sera collected from 85 dogs from different parts of Albania by indirect fluorescence antibody test (IFAT) and found that 49.4% of them had antibodies against *B. garinii*.

In the late 1990s, Çekani and colleagues [22] tested 1656 serum samples collected from sheep, goat and cattle in lowland and mountainous areas of 20 districts in Albania for *Coxiella burnetii*-specific antibodies by enzyme-linked immunosorbent assay (ELISA). In animals, this disease is mostly spread by direct contact with the pathogen after abortion, but in some cases, it can also be transmitted by infected ticks [23]. In total, 9.1% (151/1656) of the serum samples tested positive. The highest seroprevalence was observed in sheep from Permet district 45% (9/20), followed by goats from Elbasan district 39.2% (11/28) and cattle from Fier district 24.0% (6/25) [22].

CCHFV was first reported in Albania in 1986, and since then new cases have occurred almost every year, mostly in the north-eastern part of the country [24]. During the outbreak of 2001, eight CCHFV cases and a person-to-person infection were also documented. Specific immunoglobulin G (IgG) and immunoglobulin M (IgM) antibodies to CCHFV were detected in all patients except one using serology (IFAT) and molecular (PCR) methods [25]. Phylogenetic analysis has shown that the Albanian CCHFV strain from humans clusters together with other European strains.

From 2003 to 2006, 34 samples from Albanian patients suspected to be infected with CCHFV were tested for this virus by serology and PCR, and the agent was detected in 38.2% of the samples [26]. Ticks collected from cattle grazing in the endemic areas of Kukës and Has, Albania during 2003–2005 were tested for the presence of CCHFV RNA by reverse transcription PCR (RT-PCR), while serum samples collected from goats, cattle, hares and birds were tested for the presence of specific IgG antibodies [27]. In that study, of the 31 pools of ticks tested, each pool consisting of four female *Hyalomma* spp., one pool (3.2%) was found to be positive for CCHFV RNA [27]; in another study on ticks collected in eight prefectures of Albania during 2007–2014, CCHFV was detected in 30 out of 215 pools of ticks (13.9%) [28]. In the latter study, CCHFV lineage Europe 1 was characterized in 6.7% of *H. marginatum* pools collected in the Kukës prefecture in the Northern part of Albania, while CCHFV lineage 2 was detected in Berat and Gjirokastër prefectures (in southern Albania) [28]. Forty-two avian species and four hares collected in 2005 from villages in Kukes tested negative for CCHFV antibodies [27]. In sera samples collected from cattle across 10 Albanian districts in 2013, Lugaj et al. [29–31] reported the presence of CCHFV-specific IgG antibodies in 16 out of 337 samples (4.74%) using ELISA. In a subsequent study, Kadriaj et al. [32] reported a seroprevalence of 67.1% in cattle, with the highest positivity rate found among cows (88.3%) from the endemic areas.

## Bosnia and Herzegovina

### Ticks

According to Muftić [14], Neumann’s discovery of *Hy. aegyptium* in 1910 [13] represents the first written record of a tick species in Bosnia and Herzegovina. Subsequently, various researchers, applying different guidelines and methods of species identification (taxonomic keys) to investigate tick species in the country, were able to confirm the presence of a range of tick species, including *I. ricinus*, *I. hexagonus*, *I. canisuga*, *I. vespertilionis*, *Hae. punctata*, *Hae. sulcata* (syn. *H. cholodkowsky*), *Hae. inermis*, *D. reticulatus*, *D. marginatus*, *D. silvarum*, *Rh. bursa*, *Rh. sanguineus* s.l., *Rh. annulatus* (syn. *Bo. annulatus* and *Bo. calcaratus*), *Hy. scupense* (syn. *Hy. detritum*), *Hy. aegyptium*, *Hy. marginatum* (syn. *Hy. savignyi*), *Hy. excavatum* and *Hy. rufipes*. However, some of these species were most likely misidentified, such as *D. silvarum* (which only exists in Asia), *Hy. aegyptium* and *Hy. excavatum* (Table 1). A comprehensive systematic investigation of ticks was conducted as part of a doctoral thesis in 2004, involving the collection and determination of 10,050 ticks from six regions of Bosnia and Herzegovina [33, 34]. The presence of *I. ricinus*, *I. hexagonus*, *Hae. punctata*, *D. marginatus*, *D. reticulatus*, *Rh. bursa*, *Rh. sanguineus* s.l., and *Hy. marginatum* was confirmed. Furthermore, the study showed that *I. ricinus* and *D. marginatus* are the most dominant tick species in the country, constituting 70.5% and 11.5% of the collected specimens, respectively.

The most recent study of tick species in Bosnia was conducted between 2017 and 2020. A total of 6319 ticks from the family Ixodidae were collected from 96 sites across the country. Most of these ticks were sampled from various animal species, including domesticated animals such as dogs, cats, sheep, goats, horses and poultry, as well as wild hosts, such as the chamois. Only a small number of ticks (*n* = 126) were collected from the ground vegetation. The presence of seven tick species was confirmed: *I. ricinus*, *Hae. punctata*, *D. marginatus*, *D. reticulatus*, *Rh. bursa*, *Rh. sanguineus* s.l. and *Hy. marginatum* [35].

### Tick-borne pathogens

There is a lack of comprehensive data on the prevalence and distribution of zoonotic TBPs in Bosnia and Herzegovina. In a first study conducted in the central Bosnian region in 1976, the peripheral blood smears of 39 cattle were examined, with *Babesia divergens* found in 28 (71.8%) of the blood smears [36]. The infection was confirmed by microscopic examination and molecular screening of two samples and by the detection of DNA in two engorged *I. ricinus* female ticks removed from these animals [37].

Subsequent molecular studies carried out on wild carnivores (wild cats and red foxes) did not show the presence of zoonotic *Babesia*, *Anaplasma*, *Ehrlichia*, *Neoehrlichia*, SFG rickettsiae and *Bartonella* species in the tested blood and spleen samples [38, 39]. In 2018, blood samples were collected from 408 domestic dogs living under different conditions (free-roaming, client-owned, sheltered) and tested by a microfluidic real-time PCR assay for 43 different vector-borne pathogens [40]. Among the TBPs present, *Anaplasma phagocytophilum* and *Anaplasma platys* were detected in 11 (2.7%) and one (0.2%) of the dogs, respectively. In 2022, 903 blood samples from stray dogs were screened using the SNAP 4Dx Plus test and real-time PCR (qPCR) to detect antibodies against *A. phagocytophilum*/*A. platys* and *Ehrlichia canis*/*Ehrlichia ewingii* [41]. Out of 903 samples, 187 (20.7%) showed antibodies against *A. phagocytophilum*/*A. platys* and/or *E. canis*/*E. ewingii*. The highest seroprevalence (20.4%) was for *A. phagocytophilum*/*A. platys*, while two dogs had antibodies against *E. canis*/*E. ewingii* and one dog had antibodies against both. Among the 187 seropositive dogs analyzed by qPCR, 48 (25.7%) were positive for Anaplasmataceae, with 45 (24%) testing positive for *A. phagocytophilum* and one for both *A. phagocytophilum* and *A. platys*. Two Anaplasmataceae-positive samples yielded negative results in species-specific PCR analyses.

The first epidemiological study in humans was conducted in 1989 by Punda-Polić et al. [42], who determined the prevalence of antibodies against *Rickettsia* in blood samples from individuals residing in the north-western part of Bosnia and Herzegovina. Of 231 sera samples tested for *Rickettsia* using the complement fixation test (CFT), 61.5% were positive for *R. typhi*, 4.3% for *R. prowazekii*, 1.7% for *R. conorii* and 19% for *Coxiella burnetii*. In comparison, of the 183 serum samples tested for *Rickettsia* using IFAT, 37.7% were positive for *R. typhi*, 1.6% for *R. conorii* and 22.4% for *C. burnetii*.

Regarding bacteria from the *B. burgdorferi* s.l. complex, Lasić et al. [43] found 32 (66.7%) PCR-positive *I. ricinus* ticks out of 48 collected from human patients from the Sarajevo region. Another molecular screening revealed the presence of six pathogens (*Rickettsia monacensis*, *R. helvetica*, *R. raoultii*, *R. slovaca*, *A. phagocytophilum*, and *Francisella tularensis* subsp. *holartica*) of public health concern. These pathogens were detected in 19 (21.8%) of the 87 ticks belonging to the species *I. ricinus*, *D. reticulatus* and *D. marginatus* collected from pastures and wooded habitats by flagging the vegetation near human settlements and a military training camp, in south-western Bosnia and Herzegovina. None of the tested ticks were positive for *B. burgdorferi* s.l. [44].

However, in another study on pathogens in ticks in Bosnia and Herzegovina that was conducted in 2006, *I. ricinus* ticks were collected and screened to detect TBEV [45]. The authors successfully isolated three strains of TBEV from an adult *I. ricinus* tick (Bosnia-3, Bosnia-9, Bosnia 12).

Although the vector of CCHFV, *Hy. marginatum*, was reported in the country almost 100 years ago [16], the first report of animal exposure to this deadly virus came in 2022 [46]. In that study, the authors tested 176 randomly selected sheep from the Gacko and Nevesinje regions (Herzegovina) for anti-CCHFV antibodies using multi-species double antigen ELISA and IFAT. The antibodies were detected in 17 (9.6%) animals using the ELISA test, while IFAT confirmed positivity in 13 out of the 17 latter samples. The most recent study on CCHFV was conducted by Goletić et al. [47]. Out of 760 ticks collected from the tested animals, 110 were analyzed individually, and all yielded negative results; the remaining 650 ticks were divided into 90 pools, and only one pool, consisting of three male *H. marginatum* ticks, tested positive for viral RNA. In the same study, no viral RNA was detected in 206 bovine blood samples using RT-qPCR. Additionally, the overall seroprevalence of CCHFV in the cattle population was estimated to be 14.9% by serological methods.

## Croatia

### Ticks

The Republic of Croatia has a great wealth of biological and landscape diversity, with a continental and Mediterranean climate. Despite its small size, and crescent-shape feature, its geographical position makes it suitable for hosting a high diversity of tick species. Since the first studies on ticks almost 100 years ago, 23 valid species have been confirmed: *I. ricinus*, *I. hexagonus*, *I. canisuga*, *I. vespertilionis*, *I. kaiseri*, *I. frontalis*, *I. arboricola*, *I. gibbosus*, *I. trianguliceps*, *Hae. inermis*, *Hae. punctata*, *Hae. sulcata*, *Hae. concinna*, *Hae. parva*, *Hae. erinacei*, *D. marginatus*, *D. reticulatus*, *Rh. bursa*, *Rh. sanguineus* s.l., *Rh. turanicus*, *Rh. annulatus*, *Hy. marginatum*and *Hy. scupense*. There are also doubtful descriptions of species such as *D. silvarum*, *Rh*. (*Bo*.) *annulatus* and *Hy. dromedarii* (Table 1).

### Tick-borne pathogens

Well-known pathogens, such as *B. burgdorferi* s.l. and TBEV, dominate the continental and mountainous areas, while MSF is present exclusively in the central and southern Croatian Littoral. On the national level, these pathogens are included in the group of reportable diseases to the National Institute of Public Health, but several others are still not receiving sufficient attention. Pathogens such as *Borrelia miyamotoi* (relapsing fever *Borrelia*), *Neoehrlichia mikurensis*, *R. helvetica*, and *R. raoultii* have not yet been identified in humans despite their presence in animals and ticks [48–50]. Given the number of infections discovered in animals and ticks, the true number of human cases is likely to be underestimated and under-reported.

The Croatian Littoral has been designated an endemic region for MSF caused by *R. conorii*. Human cases of MSF have been reported primarily from Zadar to Dubrovnik, with a few cases also reported in Croatia’s northern coastal region (Istria, Hrvatsko Primorje). Petar Tartarglia described the first clinical case in Croatia over 90 years ago in a patient from Split [51]. The first serologically confirmed clinical cases of MSF in Croatia were found in August and September 1982 among seven residents of Split and its surrounding area using complement fixation inhibition [52]. Furthermore, *R. conorii* was isolated and molecularly confirmed for the first time in 1998 from the blood of a male patient in the Split area [53]. Morović et al. [54] documented 50 patients from Northern Dalmatia between 1986 and 1991. In the same study, 40% of 230 healthy blood donors tested positive for antibodies to *R. conorii*. Furthermore, the hemolymph test revealed that 70.2% of ticks collected from various animals and by flagging were positive for rickettsia-like infectious agents. Radulović et al. 1993 [55] used IFAT, enzyme immunoassay (EIA) and western blot (WB) to detect antibodies to *R. conorii* in serum from 477 healthy people residing along the Adriatic Coast, including Istria (42 individuals), Zadar (57 individuals), Split (103 individuals) and Dubrovnik (275 individuals). Antibodies were found in all of the study areas, with an overall prevalence of 4.2% of sera samples by the immunofluorescence assay and 5.0% of sera by EIA. Punda-Polić et al. [56] used an IFAT to detect the presence of IgG antibodies against *R. conorii* in 43.7% of samples collected from 1207 healthy citizens of central southern Croatia between 1997 and 1999. Of the 528 seropositive individuals, 30.2% were from Split, 48.3% were from suburban settlements and 52.1% were from both suburban and rural areas. Vilibić-Čavlek et al. [57] investigated the presence of IgG and IgM antibodies in febrile disease and rash patients (2009–2012) against *R. conorii* and *R. typhi*. Antibodies to *R. conorii* were found in 29% of patients, antibodies to *R. typhi* were found in 8% and 4% of patients were seropositive to both rickettsia. During the study period, 11 individuals were diagnosed with acute MSF (positive for IgM/IgG antibodies). Antibodies to rickettsiae were detected in eight serum samples out of 102 (8.16%) in a single investigation in a continental region of Eastern Slavonia, five of which were from patients with tick-borne encephalitis and three were from asymptomatic people (*n* = 62).

According to available data from the Croatian Institute of Public Health, 67 people with MSF were recorded during the 15 years between 1985 and 1999 [56], while MSF was identified in 126 individuals at Split University Hospital [58] between 1982 and 2002.

At the present time, the yearly incidence of MSF in the Split and Zadar regions ranges from one to 17 cases per year [59]. So far, the only fatal case of MSF was reported in a 58-year-old man from Zadar Dželalija [60]. In the first study aimed to detect antibodies to *R. conorii* in dogs, 30% of 62 animals from the Zadar region tested positive [54]. In a more extensive investigation [61], the IFAT was used on 194 canines from urban (49 dogs) and suburban (92 dogs) endemic areas of Southern Croatia, and on 53 canines from the non-endemic urban district of Zagreb in Northern Croatia. Antibodies to *R. conorii* were found in 48.9%, 69.6% and 20.7% of the samples, with an overall prevalence of 51%. The findings suggested that not only *R. conorii*, but also other members of the SFG could be found in non-endemic areas of Zagreb. Punda-Polić et al. [62] used a CFT to look for antibodies to *R. conorii* in serum collected from 159 different animals between June 1991 and March 1992 in the Split area. Dogs had the highest prevalence of *R. conorii* (52.9%; 27/51), followed by cattle (27.3%; 3/11), sheep (9.4%; 5/53) and goats (7.9%; 3/38), with an overall prevalence of 23.9%. In a study on small wild rodents (*n* = 94) from Gerovo and Žutica, 4.3% (2 *M. glareolus* and 2 *A. flaicollis*) showed antibodies to SFG rickettsiae and another three rodents (3.2%) (1 *M. glareolus*, 2 *A. flavicollis*) were positive for *Rickettsia* spp. by gltA-based qPCR, but determination could not be confirmed by ompB-based PCR [63]. Tadin et al. [49] failed to discover *Rickettsia* spp. DNA in 242 small wild rats collected from eight different geographical sites.

Several pathogenic rickettsiae (*R. conorrii*, *R. slovaca, R. aeshlimannii, R. helvetica, R. monacensis* and *R. raoultii*) and undetermined pathogenic rickettsiae (*R. ripcephali, R. hoogstraalii* sp. nov.) have been molecularly confirmed in ticks from different regions of Croatia.

The first such study assessed the prevalence of SFG rickettsiae in 832 ticks collected along the entire Croatian Adriatic Coast using a hemolymph test, direct immunofluorescence, antigen-capture EIA and PCR [64]. *Rickettsia*-like organisms were found in the hemolymph of 12% of *Rh. bursa* ticks, 10.6% of *Rh. sanguineus* s.l. ticks and 7.8% of *D. marginatus* ticks; four isolates were cultured and identified as *R. conorii* [64]. In another study, the rickettsiae were found in 25 (12.7%) of 197 ticks (*Hae. punctata, Hy. marginatum, Rh. bursa* and *D. marginatus*) collected from domestic animals in southern Croatia in October 2000 [65]. The prevalence in *D. marginatus* and *Hy. marginatum* ticks was 36.8% and 64.7%, respectively. None of the ticks from the *Hae. punctata* or *Rh. bursa* species were infected. *Rickettsia slovaca* was found to infect *D. marginatus* ticks, while *R. aeschlimannii* infected *Hy. marginatum* ticks. The same group of authors conducted a follow-up investigation on *Hae. punctata*, *Hy. marginatum*, *Rh. bursa, Rh. turanicus* and *D. marginatus* [66]. In that study, three out of 33 (9.1%) *Rh. turanicus* ticks were infected with *R. rhipicephali*; *D. marginatus* and *Hy. marginatum* ticks were infected with *R. slovaca* (36.8% in October 2000 and 60.0% in May 2001) and *R. aeschlimannii* (64.7% in October 2000 and 26.1% in May 2001), respectively. *Rickettsia* sp. closely related to *R. felis* was found in 23 (22.8%) ticks [67] in a study of 101 adult *Hae. sulcata* ticks collected from sheep and goats in the same region between 2001 and 2002.

Using the dragging method, 100 adult *D. reticulatus* ticks collected from various localities near Čakovec, between the Drava and Mura rivers, were tested for *R. helvetica* and *R. slovaca* [68] using qPCR. DNA from *R. helvetica* was found in 10% of *D. reticulatus* ticks, DNA from *R. slovaca* was found in 2% of *D. reticulatus* ticks and one tick was co-infected. In two studies, 1423 *I. ricinus* ticks were collected in the region around Slavonski Brod by dragging [48, 69]. Of the 1273 *I. ricinus* tested for *Rickettsia* spp. 101 (7.9%) were positive: 79 (78%) were identified as *R. helvetica*, 21 (21%) as *R. monacensis* and one (1%) as *R. raoultii* [48]. Interestingly, *R. monacensis* was the only species confirmed in the skin biopsies of *erythema migrans* from the active edge of human patients [48]. It would appear that studies aiming to confirm *Rickettsia* spp. in humans need to understand the importance of the species confirmed in ticks.

The pathogen responsible for human granulocytic ehrlichiosis (HGE), *Anaplasma phgocytophilum*, is the most intensively studied zoonotic pathogen in animals. Serological and genetic investigations on several dog groups have revealed a wide geographical distribution in both continental and littoral Croatia [50, 70–73]. Antibodies have been detected in 4.6% out of 435 and in 6.2% out of 1433 of dogs sampled from different Croatian regions [70, 71] using ELISA. In another study, only 0.3% (3/1080) of apparently healthy canines tested positive *for A. phagocytophilum* DNA [72]. Surprisingly, DNA of *A. phagocytophilum* was found in 10.5% of formalin-fixed, paraffin-embedded tissue blocks (FFPEB) from dogs that had died due to hemolytic crisis [50]. In 2017, the first case of *A. phagocytophilum* infection in a sick mare with depression, ataxia, high fever, pale mucous membranes and limb edema was reported [70]. *Anaplasma phagocytophilum* has been found in a variety of domestic and wild animal species [74, 75]. Phylogenetic analysis of a 530-bp *groEl* fragment of *A. phagocytophilum* from domestic (sheep, cows, dogs, horse) and wild animals (wild boars, brown bears, red foxes, gray wolves, stone martens, mouflon, red deer, roe deer, chamois and hare) showed the presence of three clusters (I, II, III), of which cluster I was dominant [75].

Studies on the presence and distribution of *A. phagocytophilum* in ticks and humans, in contrast to animals, are still limited. The first human case was described in 1998 in a patient from the county of Križevci- Koprivnica [73]. During a 7-year period, anaplasmosis was detected using an IFAT on paired sera samples in eight (6%) of 132 patients with febrile illnesses and a history of tick bite [76]. The disease manifested as a nonspecific, flu-like febrile illness accompanied by leukopenia, thrombocytopenia and moderately increased aminotransferase levels. From 2009 to 2012, 496 sera samples from 425 patients were analyzed for the presence of IgM and IgG anti-*A. phagocytophilum* antibodies at the University Hospital for Infectious Diseases in Zagreb. In total, 160 patients (37.6%) were found to be positive, with only three cases meeting the criteria for acute human granulocytic anaplasmosis (HGA). During the period 1998–1999, 102 people with a history of tick bites from Eastern Croatia were evaluated using an IFA. Seven (6.8%) individuals had *A. phagocytophilum* antibodies and three (2.9%) had *Ehrlichia chaffeensis* antibodies [77].

HGA is present in continental Croatian regions according to seroprevalence studies, but the DNA of *A. phagocytophilum* has never been verified in human patients. Two studies in Croatia have demonstrated the widespread distribution of *Neoehrlichia mikurensis*, a zoonotic bacterium from the *Anaplasmataceae* family, in wild animals from different regions [50, 74].

Škrabalo and Deanović [78] presented the first description of fatal human babesiosis in a 33-year-old splenectomized tailor living 10 km from Zagreb. The authors stated that the infection was caused by *Babesia bovis* based on the morphological characteristics of merozoites and the patient being a farmer. So far, *B. bovis* has not been identified as a zoonotic species, but because merozoites in microscopic images can resemble *Babesia divergens*, it may be that the infection could be attributed to *B. divergens*. This case remains Croatia's lone reported clinical case of human babesiosis. Another zoonotic *Babesia* species, *B. microti*, was identified in eight out of 242 (3.3%) small wild rodents from five distinct locations [48] and in eight out of 120 (6.6%) small wild rodents from a single area [79]. Antibodies to *B. microti* were found in a single serum samples from 102 (0.98%) individuals with a history of tick bite living in eastern Slavonia counties between 1998 and 1999 [77].

The most frequent tick-borne zoonosis in Croatia is Lyme borreliosis. Forenbacher described the first case of *erythema cronichum migrans* in 1940 (Lipozenčić and Šitum [80]), and Mohar in 1984 [81] reported the second case in patients from the northern Adriatic coast. *Borrelia burgdorferi* was initially isolated in Croatia in 1991 from the skin of a patient with erythema migrans [82]. The isolate was classified to the *B. burgdorferi* s.l. group based on electrophoretic examination of the six most significant proteins of various molecular mass (OspA, OspB, OspC, p41, p60 and p100). In the latter study, the isolate was identified as *Borrelia afzelii* [83]. By PCR, sequencing and phylogenetic analysis, *B. afzelii* was the only genospecies confirmed in the blood of 10 individuals with erythema migrans [83]. Another genospecies, *Borrelia garinii*, was isolated from the skin of a patient with erythema migrans without associated neurological and/or other signs for the first time in 2002 [84]. Tijsse-Klasen et al. obtained 44 sequences from erythema migrans samples in 2013, 42 of which were *B. afzelii*, one was *B. garinii* and, for the first time, *Borrelia bavariensis* [48].

After its initial description in Istria, the total number of recorded cases of borreliosis from 1987 to 1998 was 1922, with 79 cases (4.11%) originating from the Northern Adriatic coast [85]. The same authors raised the issue of under-reporting, stating that 170 individuals were treated at the Hospital for Infectious Diseases between 1984 and 1989, but only eight cases of borreliosis were officially documented. From 1985 to 1999, a similar group of authors recorded 2156 cases, with an average of 143.5 cases per year; 94 (4.35%) of these cases were reported from the Adriatic coast, predominantly in the northern region [86]. From 2009 to 2019, the average number of reported human cases increased to 510 ± 115.71 [87]. Several seroepidemiological studies in humans revealed a prevalence ranging from 2.4% to 47.1%. The first of these studies [88] categorized the participants into four groups: (i) the general population (healthy subjects); (ii, iii) patients without clinical evidence of Lyme disease from endemic (ii) and non-endemic (iii) regions; and (iv) forestry workers, representing the at-risk population from the endemic region. IgG antibodies were found in 9.7% of the general population (9/93) and 42.9% (30/70) of forestry workers. The authors found considerable differences between the Koprivnica endemic region (44%; 22/50) and the non-endemic city of Zagreb (8%; 4/50). Surprisingly, a low seropositivity of 4.7% was observed among Gorski Kotar forestry workers; the low prevalence (2.7%) in the general population of the coastal region was expected [89]. In eastern Slavonia, antibodies were found in 14.7% (15/102) of individuals with a history of tick bite [77]. From January 2015 to October 2017, 472 serum samples collected from patients in Istria county were analyzed for the presence of antibodies to *B. burgdorferi* s.l. [90]. The overall prevalence of IgG and IgM was 10.8% and 13.3%, respectively, in the screening test; the prevalence of IgG and IgM antibodies was 9.9% and 12.7%, respectively, using immunoblot [77].

A molecular study on 124 *I. ricinus* ticks collected by dragging in May 1995 from five locations in northern Croatia (45°5′ to 46°5′N and 15°5′ to 17°E) revealed the presence of four species of the *B. burgdorfer*i s.l. complex with an overall prevalence of 45%, including *B. afzelli, B. garinii, B. valaisiana* (group VS116) and *B. burgdorferi* sensu stricto (*B. burgdorferi* s.s.). A subsequent analysis revealed that 17.7% of 1432 *I. ricinus* samples were positive for *Borrelia* [48]. Ticks were gathered by dragging between March and June 2011 at three distinct sites located 15 to 20 km east, north and west of Slavonski Brod. Six *Borrelia* species were identified in ticks: *B. afzelii, B. garinii, B. burgdorferi* s.s., *B. valaisiana, B. lusitianae* and *B. spielmanii*. In the same study, of the 44 sequences acquired from erythema migrans, 42 were *B. afzelii*, one was *B. garinii* and one was *B. bavariensis* [48].

Molecular examinations conducted on small wild rodents revealed the presence of *B. afzelli* and *B. miyamotoi* in 2.0% (5/242) and 3.7% (9/242), respectively, of the animals examined [49]. In 2008, Turk et al. discovered *B. afzelii* in 8.88% (4/45) of fat dormouse (*Glis glis* L.) in the Gorski Kotar region [91]. Antibodies to *B. burgdorferi* were identified in 21.4% (9/42) of roe deer sera and in 33.3% (3/9) of hare sera in a seroepizootiologic investigation of wildlife and domestic animals from north-western Croatia assessed by inhibition ELISA, while sera of wild boars (*n* = 10), cattle (*n* = 103) and dogs (*n* = 13) were negative for these antibodies [92]. Dogs were also studied for seroprevalence in other studies; using ELISA, Turk et al. [93] observed a seroprevalence of 5% (6/120) in serum from 120 seemingly healthy dogs in Zagreb. In addition, Mrljak et al. [70] discovered antibodies in three of 435 (0.69%) apparently healthy dogs, whereas Jurković et al. [71] discovered an even lower overall prevalence of 0.69% (3/435) in three different groups of dogs. Additional studies are needed to understand the distribution and prevalence of *B. burgodorferi* s.l. in the non-endemic coastal region of Croatia.

Bhanja virus (BHAV) is a tick-borne bunyavirus that was initially discovered in Croatia in 1974 from *Hea. punctata* ticks collected from sheep on Brač Island [94]. The first human infection was reported in the same year, although it was not confirmed until 1980 [95], and in 1977 there was a report of two laboratory personnel who got sick while working with the BHAV [96]. The highest BHAV prevalence was found in serological investigations on Brač Island where the initial case was discovered: BHAV antibodies were found in 31.54% of the 875 sera tested from residents of the island between 1975 and 1977 [97]. A lower prevalence was found on other islands of Croatia: Hvar (1% of 512 sera samples), Mljet (3.7% of 728 sera samples), northern Dalmatian islands (9.4%) and northern Croatia islands (6.5% of 1025 sera samples) [96]. Some 40+ years after these initial studies, Vilibić-Čavlek et al. [99] investigated the prevalence of BHAV in 254 individuals with neuroinvasive disease of unknown etiology from April 2017 to October 2021 using RT-qPCR to evaluate the cerebrospinal fluid (CSF) and urine samples and a viral neutralization test on sera samples. BHAV neutralizing antibodies were discovered in the CSF samples from two patients and in the sera from 51 patients, with an overall prevalence of 20.8%; however BHAV RNA was not found in any samples. The authors concluded that further studies are needed to determine the prevalence and clinical significance of this neglected arbovirus in the Croatian population.

TBEV has been endemic in the northwestern continental section of Croatia for many years, and in recent years, this virus has been endemic in the continental region, with tiny foci in the Middle and South Adriatic regions [100]. The first case of human TBEV infection was identified serologically in the autumn of 1953 in a patient with acute meningoencephalitis from the village of Stara Vas (north-east of Zagreb) [101]. Following this first recorded case, sporadic cases were reported primarily to the east and north of Zagreb, with seven persons found to be ill in Stara Vas village between 1953 and 1961 [101]. In addition to new cases of TBE from the same region, new cases have been recorded from other sites, such as Zelina, Dugo Selo, Zlatar, Klanjec and districts south of the Sava (Velika Gorica and Jastrebarsko), among patients treated at Zagreb Hospital [102]. From 1961 to 1963, Zorinc [103] documented 14 cases of TBE from the rural area near Križevci and Koprivnica. At the same time, Ribarić-Vince et al. [104] reported the first two cases in Zadar (Middle Adriatic region) in 1959 and 1961, while the first two cases in Pula (Northern Adriatic region) emerged in 1962 and 1963. Between 1961 and 1973, patients with TBE were documented in Pakrac (Central Croatia), Istria (Northern Adriatic) and Zadar (Middle Adriatic region) [104]. In a 1962 investigation, Vesenjak-Hirjan et al. [105] found antibodies in 22.43% (24/107) of humans tested and 56.09% (82/46) of sheep tested in the village Nadsela on Brač Island. A serologically proven case of TBE was discovered in the coastal region of Dubrovnik in 1966 for the first time [101]. Up to 1976, around 40 clinical TBE cases were documented in Croatia [101]. From 1953 to the end of 1973, 873 cases were serologically confirmed in provincial hospitals throughout Croatia [102]; the same authors reported an additional 165 patients with TBE based on clinical and epidemiological data but all of these were serologically negative. It is worth noting that 89% of people reported tick bites, with 780 of the tick bites (89.34%) occurring between May and August [106]. Raos reported 23 occurrences of TBE in the municipalities of Donji Miholjac and Slavonska Orahovica (Eastern Croatia) between 1964 and 1985 [107]. During the 1981–1984 test period, the prevalence of antibodies in forest workers, based on the inhibition of hemaglutination, was 19.1% (26/136) [107]. From 1961 to 1964, Vesenjek-Hirjan et al. [108] conducted a serological study in 24 households, comprising 97 people, 86 cows and 24 horses, using the hemagglutination inhibition test (HI), CFT and neutralization test (NT) in the village of Stara Ves, where seven cases occurred between 1952 and 1961. TBE antibodies were detected in 32.99% (HI), 32.43% (CFT) and 32.14% (NT) of humans using these different methodologies; Statistically significant differences were found in animal experiments (horse: 91.67% [HI], 100% [CFT] and 86.96% [NT]; cows: 51.16% [HI], 40.58% [CFT] and 32.14% [NT]). The same methodologies were used to study TBE prevalence in Stara Ves once again in 1972 (80 humans, 55 cows and 24 horses) [109]. In humans, the prevalence was 42.50% (HI), 32.50% (CF) and 47.50% (NT). Statistically significant differences were found in the analyses on animals (horse: 62.50% [HI], 45.83% [CFT] and 58.83% [NT]; cows: 54.54% [HI], 1.82% [CFT], 60.00% [NT]). Based on these results, the authors determined that Stara Ves was still an active TBEV focus [109]. Vesenjak-Hirjan and Šooš [110] studied 91 sheep in the settlement of Barievići on the Adriatic coast near Zadar and found antibodies in 53.7% (58/91). Raos reported 23 occurrences of TBE in the municipalities of Donji Miholjac and Slavonska Orahovica (Eastern Croatia) between 1964 and 1985 [107]. During the 1981–1984 test period, the prevalence of antibodies in forest workers, based on the inhibition of hemaglutination (HI test) was 19.1% (26/136) [107]. The Croatian Institute of Public Health recorded an average of 246 TBE cases per year (minimum 11, maximum 37) from 1999 to 2008 [111]; from 2009 to 2021, the average number of recorded human cases per year was 24.3, with a decreasing trend beginning in 2014 [112].

Two outbreaks linked to raw goat milk and cheese consumption have been recently documented in Croatia [113, 114]. Seven members of two households, out of 10 persons exposed, were hospitalized within 3 weeks of consuming raw goat milk or cheese from the same supplier near the town of Bjelovar in April and May 2015. TBEV infections was verified in all patients using an ELISA [113]. In the second outbreak, five of the six patients with TBE consumed unpasteurized goat milk in the 2 weeks preceding the onset of symptoms. RT-PCR was used to screen milk samples from 12 goats from the suspected farm for TBEV; although no TBEV RNA was found in the milk, antibodies were found in goats, horses and dogs. Six goats from the flock tested positive for TBEV-neutralizing antibodies, indicating that raw goat milk from the farm was the cause of the outbreak.

In 1976, the first investigation on viral isolation from humans and ticks was published from the regions of Stara Ves, Medvednica, Donji Miholjac and Vinkovci [106]. In that study, a total of 5751 ticks were collected by flagging and dragging across the grass and low vegetation. In total, 10 blood samples and seven 7 CSF samples were obtained from eight patients suffering from acute illnesses. They used 30 adults and 50–100 nymphs in one pool. In total, 15 strains were recovered, one from *D. pictus* and the others from *I. ricinus*, whereas *Hae. concina* was found to be negative. The minimal infection rate was 3.8% in the northwestern region and 3% in the eastern region [106].

In a single study on small wild rodents, all 194 animals from two distinct sites (mountainous and lowland regions) in Croatia were negative for TBEV based on the IgG indirect immunofluorescence test or qRT-PCR in the first ever molecular investigation [63, 100]. The prevalence of TBEV (assuming only one tick in each positive pool was infected) in red fox ticks was 1.6% (95% confidence interval [CI] 0.7–3.5%) [115]. During 2011–2012, spleen samples were taken from 182 hunted red deer from two TBE-endemic locations in north-eastern Croatian counties. TBEV RNA was found in two of the 182 spleen samples (1.1%; 95% CI 0.3–3.0%). Based on nucleotide and amino acid sequence analysis, phylogenetic analysis showed two clusters corresponding to the European subtype TBEV [115].

Although research on ticks and TBPs in humans has a long history, several issues remain unanswered and require further investigation. We should expect more human cases and new and emerging pathogens like CCHFV in Croatia due to the diversity of tick fauna and the potential of ticks to transmit multiple pathogens. Further research using a One Health approach is required to fill up the gaps in current knowledge on TBPs in Croatia, particularly regarding diagnostics and pathogen spread in ticks.

## Montenegro

### Ticks

The first written record of tick occurrence in Montenegro dates back to 1938 when Oswald [16] reported the following species: *I. ricinus*, *Hae. punctata*, *Hae. inermis*, *D. reticulatus*, *Rh. bursa*, *Rh. sanguineus* s.l., *Rh*. (*Bo*.) *annulatus* and *Hy. scupense* (syn. *Hy. detritum*). Later reports/studies revealed additional species, namely *I. simplex*, *I. vespertilionis*, *Hae. sulcata*, *D. marginatus* and *Hy. marginatum* [3, 116]. Similar to other Western Balkan countries, the descriptions of certain tick species, such as, for example, *D. silvarum*, *Hy. rufipes* (syn. *Hy. impressum*) and *Hy. dromedarii*, is likely to be wrong since they are not native to Europe (Table 1) [3].

### Tick-borne pathogens

Montenegro is considered to be an endemic country for many TBDs. With its geographical position, together with its ecological and demographic characteristics, Montenegro represents a suitable environment for a wide range of zoonoses [116]. Although the first human case of vector-borne disease (leishmaniasis) in Montenegro was recorded in 1924 [117], data on human and animal TBDs are very scarce, and there is an urgent need for more extensive and systemic studies.

SFG rickettsiae and Q fever in Montenegro were reported for the first time in 1995/1996. Out of 657 patients who were tested in the period from 1996 to 2017, 293 had confirmed rickettsial spotted fever [118]. In another study, antibodies to *Rickettsia conorii* were detected in sera from 73.4% of dogs, while 19.3% and 1.2% of the dogs had antibodies to *E. canis* and *C. burnetii*, respectively [119]. Interestingly, in this study, *R. conorii* was more prevalent in pet dogs than in dogs from public shelters; the opposite was true for *E. canis* [119]. Q fever was reported in 158 human cases in the period from 1996 to 2017 [118]. A seroprevalence of 5.0% was observed in sheep sera collected in 2000 and 2001, which represents the only study conducted thus far on the presence of *C. burnetii* in animal reservoirs [120].

Human babesiosis was first confirmed in 2011 based on hematological and parasitological examinations. Since 2013, 12 cases have been documented in Montenegro, among which, six patients had erythema migrans that was associated with Lyme borreliosis. Coinfection with *Babesia* parasites and *B. burgdorferi* spirochetes was confirmed by ELISA, WB and PCR in 72% of the 12 patients with confirmed babesiosis [117, 121].

## North Macedonia

### Ticks

Data on tick presence, distribution and host preference in N. Macedonia are scarce, with only a few published reports. Most of the studies were conducted in the first half of the last century (1930–1960), followed by a 50-year gap in tick research, resulting in a considerable lack of information on and understanding of tick ecology. All of the tick studies carried out in N. Macedonia were based on data from ticks collected directly from the host, including cattle, sheep, goats, horses, dogs, bats and humans. The tick fauna of N. Macedonia includes 15 species of hard ticks belonging to five genera (Table 1). Historical research on the tick fauna in N. Macedonia from the 1930s onwards has been documented by Oswald [122] and updated by Angelovski [123–125] with additional studies conducted in the 1950s. These authors provided a detailed report on the tick fauna in the Skopje region where they discovered *I. ricinus*, *Hae. inermis*, *Hae. punctata*, *Hae. sulcata*, *D. marginatus*, *D. reticulatus*, *D. silvarum*, *Rh. bursa*, *Rh. sanguineus* s.l., *Rh. annulatus* (syn. *Boophilus calcaratus*), *Hy. marginatum* (syn. *Hy. savignyi*), *Hy. excavatum*, *Hy. rufipes* and *Hy. scupense* (syn. *Hy. detritum*). More than 50 years later, Pavlović et al. [126, 127] reported the presence of *I. ricinus*, *Hae. punctata*, *D. marginatus*, *D. reticulatus*, *Rh. bursa*, *Rh. sanguineus* s.l. and *Rh*. (*Bo*.) *annulatus* parasitizing sheep, cattle and dogs in the Kumanovo region, with *I. ricinus* being the most abundant species. The presence of *Hy. rufipes* is mentioned by Apanaskevich and Horak [3].

### Tick-borne pathogens

Although the presence of both pathogens and diseases with significant public health importance have been reported in N. Macedonia, the studies and the published data on TBPs circulating in this country are fragmentary. The first outbreak of CCHF in humans was recorded in the village of Chiflik, near Tetovo in 1970, with 13 confirmed human cases of which two had a lethal outcome [128]. In 1977, Gligić et al. [188] confirmed the presence of CCHV in ticks collected from the Tetovo region, thus retrospectively confirming the 1970 outbreak. Up to 2010, an additional 12 human cases were recorded [98], none of which were fatal. More than 50 years after the first outbreak, a new fatal autochthonous case of CCHFV infection in a patient from the village of Kuchica, near Shtip, and a nosocomial infection in a healthcare worker who cared for the patient were described by Jakimovski et al. [129]. Countrywide, serological studies conducted on sera collected from cattle [130], sheep and goats [131] revealed a high seroprevalence of CCHFV and widespread distribution of the virus in the country. Mertens et al. [130] reported an overall seroprevalence of 14.6% (range 0–80%) in cattle, whereas Schuster et al. [131] reported a seroprevalence of 75% (range 44-86%) and 59% (range 0%-75%) in sheep and goats, respectively.

In naturally exposed dogs, Stefanovska et al. [132] found serological evidence of *E. canis* and *Leishmania infantum* coinfection, and a seroprevalence of 18.7% for *E. canis*. In a study carried out in the city of Skopje, Atanaskova Petrov et al. [133] reported the first evidence of *E. canis* in dogs using molecular methods.

Meloska and Jankijević [134] found that 18.8% (45/240) of the tested patients were positive for anti-*Borrelia* IgG and IgM antibodies. Of all patients tested, 25.8% (62/240) reported a tick bite within 10–60 days before testing. The seroprevalence in the patients who reported a tick bite was 28.9% (13/45) [134]. Recently, Jakimovski et al. [135] observed that tick-infested individuals had lower seroreactivity against specific *Borrelia* proteins OspC and VlsE of 8.88% and 2.17%, respectively, and found TBEV-neutralizing antibodies in only one sample (2.22%) from the tick-exposed group from Skopje.

In another study, Jakimovski et al. [136] reported a case of MSF-like illness, a tick-borne disease caused by *R. sibirica mongolitimonae*, in a patient bitten by a *Hyalomma* tick in the Skopje region.

Taken together, these studies suggest that the risk of contracting TBDs is high in animals and humans bitten by ticks, thus highlighting the importance of conducting comprehensive research on ticks and TBPs in the Republic of N. Macedonia.

## Serbia

### Tick species

Ixodid tick fauna of Serbia includes 21 species belonging to five genera (Table 1). The first studies on ticks in Serbia began in the late 1930s in the southern region of the country [137]. Based on subsequent surveys, in 1979 Petrović [138] reported 14 species of hard ticks, as follows: *I. ricinus*, *I. persulcatus*, *Hae. punctata*, *Hae. sulcata*, *Hae. inermis*, *Hae. leporispalustris, D. marginatus*, *D. reticulatus* (syn. *D. pictus*), *Rh. bursa*, *Rh. sanguineus*, *Rh. annulatus* (syn. *Bo. calcaratus*), *Hy. marginatum* (syn. *Hy. savignyi*), *Hy. scupense* (syn. *Hy. detritum*) and *Hy. excavatum*. During extensive faunistic and ecological surveys of ticks throughout Serbia, Milutinović and colleagues [139–142] recorded ten species of hard ticks: *I. ricinus*, *Hae. punctata*, *Hae. sulcata*, *Hae. inermis, D. marginatus*, *D. reticulatus* (syn*. D. pictus*), *Rh. bursa*, *Rh. sanguineus*, *Rh. annulatus* (syn. *Bo. annulatus*), *Hy. marginatum* (syn. *Hy. savigny*). Recent surveys in Serbia on tick species parasitizing wild animals revealed the presence of *I. hexagonus*, *I. canisuga*, *I. kaiseri* and *Hae. concinna* in red foxes [143, 144], *I. laguri* in ground squirrels [145] and *I. vespertilionis* and *I. simplex* in bats [145].

### Tick-borne pathogens

So far, seven species of tick-borne *Borrelia* have been detected in Serbia, *B. miyamotoi* and six species belonging to the *B. burgdorferi* s.l. complex. The first detection of *B. burgdorferi* s.l. in *I. ricinus* ticks in Serbia was in 1993 in the Belgrade area [146], and in the same year, the pathogen was isolated from mice (*Apodemus flavicolis*) [147]. Since then, *B. burgdorferi* s.l. has been reported in *I. ricinus* ticks with a prevalence ranging from 10.8% to 33.2%, in dogs with a prevalence ranging from 8.1% to 26.1% and in humans with a prevalence ranging from 8.6% to 54.2% [148–157]. As of 2008, the characterization of Lyme borreliosis includes six species, namely *B. burgdorferi* s.s., *B. afzelii*, *B. garinii*, *B. lusitaniae*, *B. valaisiana* and *B. bavariensis* [158–163]. Studies by Milutinović et al. [158] and Ćakić et al. [162] demonstrated a dominance of *B. lusitaniae* in *I. ricinus* ticks over other *B. burgdorferi* species. In ticks collected from golden jackals, Sukara and colleagues identified *B. garinii* in *D. reticulatus* ticks [161]. *Borrelia lusitaniae*, *B. afzelii* and *B. valaisiana* were identified in* I. ricinus* collected from humans [163], while Sukara and colleagues reported *B. burgdorferi* s.s., *B. lusitaniae* and *B. garinii* in red foxes [12]. *Borrelia miyamotoi* was identified in 2016 in *I. ricinus* ticks collected from vegetation [160] and in 2021 in *I. ricinus* ticks collected from humans [163].

Although the first clinical description of animal babesiosis dates back to the nineteenth century, and numerous pathogens from the genus *Babesia* have since been detected in ticks and domestic and wild animals, no human cases have been documented in Serbia [164]. In 2016, Potkonjak et al. [165] confirmed the presence of the zoonotic *Babesia* species, *B. microti* (1.4%) and *B. venatorum* (2.8%) in *I. ricinus* ticks collected from vegetation or dogs. *Babesia microti* DNA was also recorded in ticks collected from golden jackals [161]. These findings were confirmed in a recent study by Banović et al. [166] using an unbiased high-throughput pathogen detection microfluidic system. Gabrielli et al. [167] also reported the presence of *B. microti* (1.9%) in dogs.

Tularemia first detected in 1958 in western Serbia (Užice) [168] and has occurred sporadically since then, with the first outbreak recorded in the Sokobanja region in late 1998 [169]. *Francisella tularensis* was first isolated in 1999, from mice of the genus *Apodemus* sampled from the area of the Rtanj mountains. The pathogen was also detected in *I. ricinus* ticks collected from vegetation, with a prevalence of 3.8% [158].

Research on SFG rickettsiae in Serbia has significantly increased in the last two decades. Using IFAT, Samardžić et al. [170] detected seroreactive human blood sera (9/134) against antigens of *Rickettsia conorii*, while Gajinov et al. [171] described tick-borne lymphadenopathy (TIBOLA) caused by *R. slovaca* in two female patients after tick bites. In a recent study, Banović et al. [172] described five confirmed, two suspected and two probable SFG rickettsiae infection cases, and reported that molecular analysis showed intra-species variability in *R. helvetica* strains infecting humans in Serbia. Radulović et al. [173] reported the first detection of *R. helvetica* (7.7%) and *R. monacensis* (15.4%) in *I. ricinus* ticks. Similar findings were published 2 years later by Tomanović et al. [174] and Li et al. [175] in 2019. Potkonjak et al. [165] confirmed the presence of *R. monacensis* in *I. ricinus* ticks and detected *R. raoultii* and *R. massiliae* in Serbia for the first time. In more recent studies, Banović et al. [163, 166, 172] confirmed the presence of the above-mentioned rickettsiae and additionally detected *R. slovaca* and *R. aeschlimannii* (3%) in ticks. However, data on the presence of tick-borne rickettsiae in animals are scarce. Using IFAT in a limited cross-sectional epidemiological study, Spasojević-Kosić et al. [176] reported a high seroprevalence (44.83%) of antibodies against *R. conorii* antigens in dogs; however, the data obtained by IFAT have limited value due to the significant cross-reactivity among *Rickettsia* spp. Fournier et al. [177] detected Astrakhan fever rickettsia in four *Rh. sanguineus* s.l. specimens, three of which were collected from dogs and one from military personnel.

A number of studies performed during the last 20 years have focused on determining the presence of *Anaplasma phagocytophilum* on ticks, dogs and red foxes. During the period 2001–2004, *A. phagocytophilum* was detected by PCR in *I. ricinus* ticks with a prevalence of 13.9% [158, 178]. From 2007 to 2009, *A. phagocytophilum* was detected in *D. reticulatus* (1.9%), *Hae. concinna* (2.9%) and *I. ricinus* (3.7%) [174]. During the period 2008–2013, Potkonjak et al. [179] reported a seroprevalence of 15.5% in dogs by IFAT and 0.9% in golden jackals using PCR [161]. In 2014, 1.4% of *I. ricinus* ticks tested positive for *A. phagocytophilum* DNA [165], and in 2019 the prevalence was fourfold higher (6%) in *I. ricinus* collected from humans [163].

In dogs, a single case of *E. canis* was molecularly confirmed in 2018 [180]. Also, in humans, two cases of human monocytic ehrlichiosis were diagnosed by IFAT [181, 182].

Potkonjak et al. [165] discovered *Neoehrlichia mikurensis* in *I. ricinus* ticks (4.2%) collected from vegetation. In a subsequent study, Banović et al. [163] detected *N. mikurensis* in ticks with a prevalence of 1.1%.

Many studies have been performed on the seroprevalence of Q fever in cattle and sheep, but confirmation of the pathogen has been made in only a few of these. In 2007–2009, *Coxiella burnetii* was identified in *D. reticulatus* (3.8%), *Hae. concinna* (28.6%) and *I. ricinus* (62.9%) by PCR [174]. In 2011, presence of the pathogen was confirmed by PCR in 10.5% of *Rh. sanguineus* s.l. ticks collected from stray dogs [183]. In a study on red foxes carried out from 2010 to 2016, *C. burnetii* was not confirmed in any of the samples by PCR [12].

TBEV was first isolated from ixodid ticks from the area of Pešter, Raška in 1972 [184]. During the period 2014–2015, antibodies against TBEV were confirmed in humans (0.37%), dogs (17.5%), horses (5%) and cattle (2.5%) using ELISA, but not in goats [185]. In the same study, 12.5% wild boars and 2.5% roe deer were found to harbor antibodies, while TBEV was molecularly confirmed in *I. ricinus* ticks collected from two localities. In 2018, TBEV was confirmed in three humans [186], and then again in 2020 with a seroprevalence of 13.3% using IFAT [187].

The first to study CCHFV in Serbia was Gligić et al. [188], who obtained two isolates of CCHFV from *Hy. marginatum* (syn. *Hyalomma plumbeum*) and *I. ricinus* ticks. Two human cases, of which one was fatal, were reported more than two decades later [189].

## Conclusions and perspectives

According to the available data, 32 ixodid tick species belonging to five genera have been recorded in the Western Balkans. However, some older studies reported the occurrence of ticks that are not present in southern Europe, such as *I. persulcatus* (north-eastern Europe and northern Asia), *Hae. leporispalustris* (North America), and *D. silvarum* (mostly Asia), and these were likely mistaken for other members native to the region. The natural distribution of *Hy. rufipes*, *Hy. dromedarii*, and *Hy. aegyptium* includes Africa and part of Asia, and the reports of their presence outside these regions are believed to be a consequence of the dissemination of immature stages by migratory birds. Still, these ticks have not established populations in the Western Balkans [3]. Despite the first studies on ticks being done a century ago, more current data, including DNA confirmation of species, are still unavailable from several countries. Furthermore, there is a noticed shortage of continued studies on tick activity and geographic distribution in response to environmental and climate changes.

Unlike mosquito-borne diseases, authorities are not yet sufficiently aware of TBDs. Most of the ticks reported here are known vectors of zoonotic pathogens, some of which are autochthonous in the countries of Western Balkans with highly variable incidence; however, under-reporting is potentially extensive. Thus, there is a need for integrated surveillance and reporting, and we advocate that authorities in these countries should encourage research and national monitoring programs by providing financial support. Molecular/direct pathogen confirmation in human patients remains a big issue in almost every Balkan country, and is of particular concern when faced with emerging, but still neglected pathogens detected in ticks and animal reservoirs. International and interdisciplinary collaborations, including the exchange of expertise, experience and resources, should also be encouraged as such efforts can facilitate research that has the potential to fill in the gaps in our knowledge on ticks and TBDs. The importance of international collaborations is also reflected in the current review article, which represents an outcome of a workshop on vectors and vector-borne disease held in 2013 in Germany. Almost all authors participated in the workshop and, since then, have actively collaborated on various research topics dealing with arthropod vectors and the pathogens they transmit. Additionally, improving curricula in teaching TBDs in human and veterinary medicine from an early start is fundamental for defining effective control strategies in the light of a One Health concept. The creation of integrated web-based platforms and workshops for the continuing education of veterinarians, physicians and other specialists in various fields, establishment of an expertise network and the publication of articles written in local languages so that practitioners become aware of the actual situation in the countries and the region are also highly encouraged.

## Data Availability

Not applicable.

## Abbreviations

CCHFV: Crimean-Congo hemorrhagic fever virus
CFT: Complement fixation test
ELISA: Enzyme-linked immunosorbent assay
IFAT: Indirect immunofluorescence test
HI: Hemagglutination inhibition assay
MSF: Mediterranean spotted fever
qPCR: Real-time PCR
RLBH: Reverse line blot hybridization
RT-PCR: Reverse transcription PCR
SFG: Spotted fever group
s.l.: Sensu lato (in a broad sense)
s.s.: Sensu stricto (in a strict sense)
TBD: Tick-borne disease
TBEV: Tick-borne encephalitis virus
TBP: Tick-borne pathogen
TIBOLA: Tick-borne lymphadenopathy
WB: Western blot
